# Case Report: Evaluation of *COL4A5* non-canonical splicing variants in two families

**DOI:** 10.3389/fmed.2025.1611334

**Published:** 2025-09-29

**Authors:** Chee Teck Koh, Tina Si Ting Lim, Alwin Hwai Liang Loh, Yan Fei Ng, Jia Liang Kwek, Perry Yew Weng Lau, Hui-lin Chin, Jun Li Ng, Mya Than, Irene Yanjia Mok, Cynthia Ciwei Lim, Kay Yuan Chong, Jason Chon Jun Choo, Hui Kim Yap, Kar Hui Ng, Yaochun Zhang

**Affiliations:** ^1^Department of Paediatrics, Yong Loo Lin School of Medicine, National University of Singapore, Singapore, Singapore; ^2^Khoo Teck Puat-National University Children’s Medical Institute, National University Health System, Singapore, Singapore; ^3^Department of Anatomical Pathology, Singapore General Hospital, Singapore, Singapore; ^4^Department of Renal Medicine, Singapore General Hospital, Singapore, Singapore; ^5^Medicine Academic Clinical Programme, Singapore General Hospital, Singapore, Singapore

**Keywords:** Alport syndrome, COL4A5, splicing variants, minigene assay, variant interpretation

## Abstract

**Introduction:**

Alport syndrome is one of the most prevalent monogenic kidney diseases, resulting from the defects in *COL4A3*, *COL4A4*, and/or *COL4A5* genes. Interpretation of non-canonical splicing variants can be challenging. This study aimed to resolve two variants at non-canonical splice sites in the *COL4A5* gene using multiple modalities.

**Methods:**

Exome sequencing was performed on two families suspected of having Alport syndrome. Intronic splice site variants in *COL4A5*, which have been reported in the literature, were identified: c.1032 + 4A > G in one family and a four-nucleotide deletion, c.1032 + 3_1032 + 6delAAGT, in the other. To clarify the pathogenicity of these variants, several analyses were performed: familial co-segregation analyses in relation to comprehensive phenotyping of family members, immunofluorescence analysis of kidney biopsy specimens to evaluate collagen IV α5 staining patterns, and minigene splicing assay to assess the impact on pre-messenger RNA (mRNA) splicing.

**Results:**

The family studies demonstrated co-segregation of the variants among members with characteristic phenotypic features. The immunofluorescence analysis of the kidney biopsy samples displayed aberrant collagen IV α5 staining patterns. The minigene splicing assay revealed that both variants caused exon 18 skipping in the *COL4A5* gene, resulting in truncated transcripts.

**Conclusion:**

The study demonstrated a multi-faceted approach to improve the diagnostic accuracy and clinical utility of genetic testing for Alport syndrome.

## Introduction

Alport syndrome is one of the most common monogenic kidney diseases (1). It is caused by defects in the *COL4A3*, *COL4A4,* and/or *COL4A5* genes, which encode the α3, α4, and α5 chains of collagen IV, respectively. Each of these chains intertwines to form a triple helix, which interconnects to form the glomerular basement membrane (GBM). Pathogenic variants in these three genes may occur as missense, deletion, insertion, nonsense, or splice site variations, resulting in the loss of protein function or an altered protein morphology (2).

There are four forms of Alport syndrome: autosomal dominant Alport syndrome (ADAS), autosomal recessive Alport syndrome (ARAS), X-linked Alport syndrome (XLAS), and digenic Alport syndrome. Individuals with Alport syndrome can develop microhematuria in early life, and this may progress to proteinuria and subsequently chronic kidney disease (CKD). Some patients do not have classical phenotypes. Instead, they present with cystic kidneys (3–5), early-onset hypertension, or “CKD of unknown cause” (1). Patients with ARAS or XLAS may develop neurosensory hearing loss and/or ocular lesions. Characteristic electron microscopic features of Alport syndrome include splitting, scalloping, lamellation, and a basket-weaving appearance of the GBM in the kidney. Immunofluorescence analysis of collagen IV α3, α4, or α5 chains may be useful in confirming the diagnosis of Alport syndrome, as decreased or abnormal staining patterns of collagen IV α3, α4, and α5 can occur, depending on the genotype (6). Early and aggressive blockade of the renin–angiotensin–aldosterone system may delay kidney failure by two decades or more (7, 8). The earlier the treatment is started, the greater the impact on kidney survival (7). Alport syndrome is largely diagnosed through kidney biopsies or genetic testing. However, kidney biopsies are traditionally not clinically indicated in the early stage of the disease when only isolated microhematuria or mild albuminuria is present. Moreover, the classical histological changes associated with Alport syndrome are often not present in the early stages of the disease. Therefore, the early diagnosis of Alport syndrome—crucial for significantly improving kidney survival, primarily relies on genetic testing.

The interpretation of variants of uncertain significance (VUSs) remains a significant challenge in genomic medicine. Several additional approaches may be employed to facilitate the reclassification of a VUS into either pathogenic or benign categories. The selection of these strategies is depends on the specific characteristics of the variant, the clinical and familial context, and the available evidence from the scientific literature and genomic databases. Potential strategies may include comprehensive phenotyping of the proband or affected family members, segregation analysis through variant-specific testing in relatives, and functional studies (9, 10). Nevertheless, the implementation of these investigations in routine clinical practice is often limited by feasibility constraints or may yield inconclusive results.

Functional studies performed in the molecular laboratory may provide further information that can help re-classify the VUS, particularly when conventional clinical approaches are unfeasible or inconclusive. Specifically, the minigene splicing assay has emerged as a robust method to evaluate the effects of VUSs located at non-canonical splice sites, which are particularly challenging as there is no established consensus or approach (11–13).

In this report, we describe two unrelated families, each presenting with clinical manifestations consistent with Alport syndrome. Both families harbored a distinct non-canonical intronic splice site variant in the *COL4A5* gene, which had been classified as VUSs by commercial sequencing laboratories. We aimed to resolve these two VUSs by integrating additional lines of evidence, including the minigene splicing assay, in accordance with the guidelines established by the American College of Medical Genetics and Genomics (ACMG)/Association for Molecular Pathology (AMP) (14).

## Case description

### Family A

**Figure 1:**
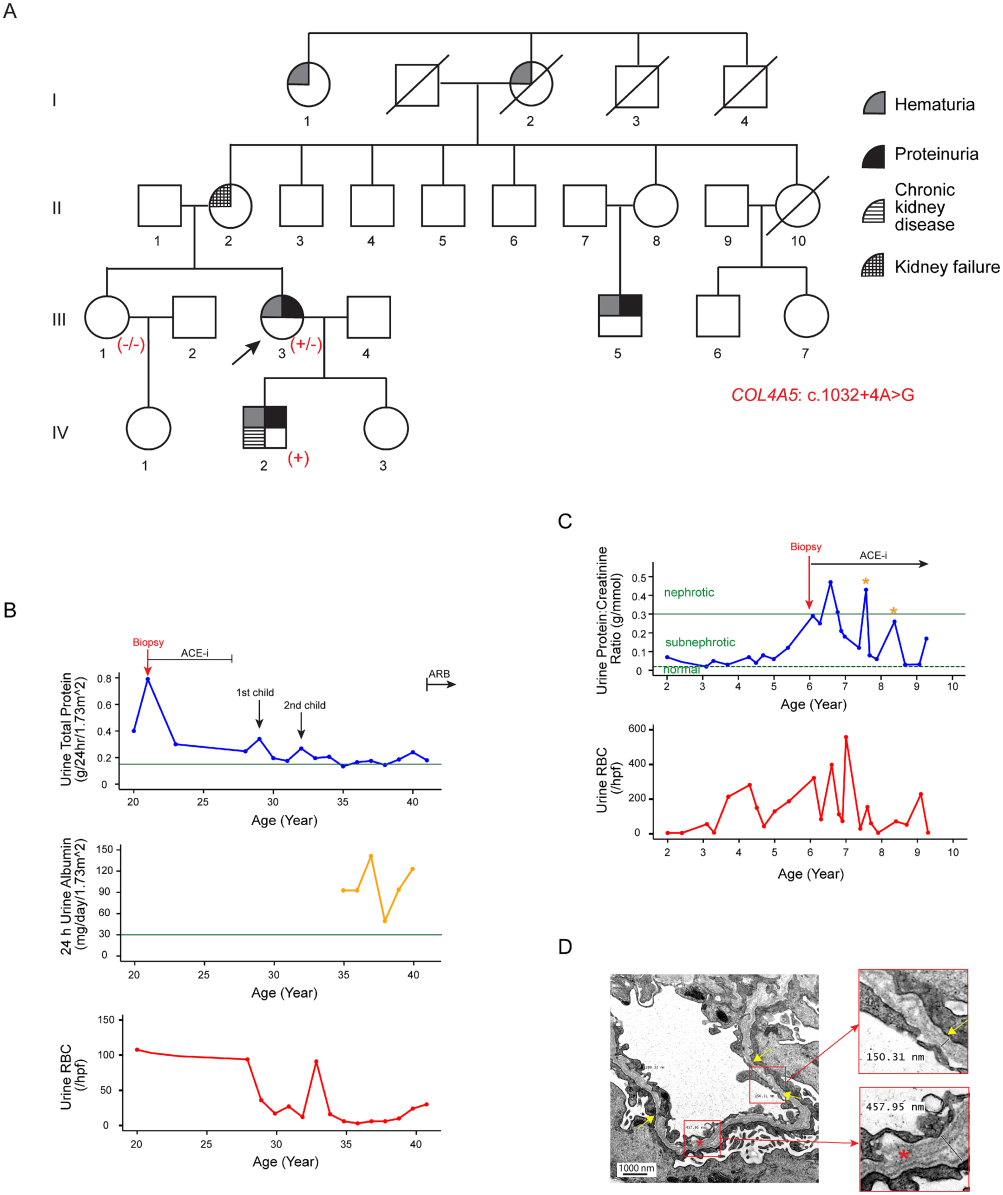
Clinical features of Family A associated with the variant c.1032 + 4A > G in *COL4A5*. **(A)** Pedigree of the affected family. Family members are denoted by generations I-IV. The arrow marks the proband; a slash strikethrough indicates deceased individuals. Genotypes are shown as −/− for non-carriers, +/− for heterozygous female individuals, and + for hemizygous male individuals carrying the variant. **(B)** Disease progression in the proband (III-3). The 24-h urinary total protein (g/day/body surface area 1.73m^2^), 24-h urinary albumin (mg/day/body surface area 1.73m^2^), and urine red blood cell count (RBCs/hpf) are displayed against age in years. The green horizontal lines in the upper and middle panels indicate the cutoff values for KDIGO categories A3 (24-h urine total protein, 0.15 g/day) and A2 (24-h urine albumin, 30 mg/day) albuminuria, respectively. **(C)** Disease progression in the proband’s son (IV-2). The urine protein-to-creatinine ratio (g/mmol) and urinary red blood cell count (RBCs/hpf) are displayed against age in years. He exhibited persistent microscopic hematuria and subnephrotic proteinuria from the age of two. ACE-i therapy was initiated at age six following a kidney biopsy suggestive of Alport syndrome. The two yellow asterisks indicate episodes of viral infections associated with transient worsening of proteinuria. The green horizontal dotted and solid lines represent the cutoff values for sub-nephrotic (urine protein:creatinine ratio: 0.02 g/mmol) and nephrotic (urine protein:creatinine ratio: 0.3 g/mmol) proteinuria, respectively. **(D)** Representative electron microscopy images from the kidney biopsy of the proband’s son (IV-2). Electron microscopy revealed significant variability in GBM thickness, with alternating regions of thinning (~ 150 nm; yellow arrows and upper insert) and thickening (~ 460 nm; lower insert). The normal GBM thickness for a 6-year-old male individual ranges from 198 to 371 nm (15). The lamellated and irregular morphology of the GBM (red asterisk and lower insert) is highly indicative of Alport syndrome. In addition, the overlying podocyte foot processes were blunted and partially effaced.

The proband (III-3; Figure 1A), a 41-year-old Chinese woman, was first noted incidentally to have persistent microscopic hematuria at the age of 4. Urinalysis showed 40 red blood cells (RBCs) per high-power field (hpf), 45% of which were dysmorphic. She had normoalbuminuria, a normal estimated glomerular filtration rate (eGFR), and a negative autoimmune screen. The microhematuria persisted into adulthood, with urine RBC counts reaching up to 130 per hpf. By the age of 21, she had developed subnephrotic albuminuria (Kidney Disease: Improving Global Outcomes [KDIGO] category A3). Following a kidney biopsy (see Histological examination below), angiotensin-converting-enzyme inhibitor (ACE-i) therapy was initiated but subsequently discontinued at age 27 due to reproductive considerations. During her two pregnancies, her proteinuria worsened transiently, peaking at 0.35 g/day/1.73m^2^. ACE-i treatment was reinstated after the pregnancies, resulting in stabilization of her proteinuria. At present, her eGFR remains normal, and the urine protein-to-creatinine ratio is subnephrotic at 0.04 g/mmol (normal <=0.02 g/mmol) with an angiotensin II receptor blocker (ARB) treatment (Figure 1B). A hearing check at age six was normal and has not been repeated since.

The proband’s mother (II-2) was diagnosed with hypertension and probable glomerulonephritis during pregnancy. The hypertension persisted after pregnancy, but she discontinued treatment. Eventually, she developed kidney failure by the age of 60, requiring kidney replacement therapy.

The proband’s son (IV-2) displayed persistent microscopic hematuria (up to 56 RBCs/hpf) and subnephrotic proteinuria (urine protein-to-creatinine ratio 0.07 g/mmol) from the age of two (Figure 1C). His eGFR was normal at the initial presentation. He was given ACE-i from age six. His CKD had progressed to KDIGO stage G2A2 by the age of 9 years. A hearing assessment at age six was normal.

The proband’s maternal male cousin (III-5) first presented at the age of 33 with incidental microscopic hematuria during health screening. Urine analysis revealed 10 RBCs/hpf, 60% of which were dysmorphic, along with normal albuminuria and preserved eGFR. Kidney ultrasound scans were unremarkable. He defaulted on follow-up but re-presented at the age of 44 with A2 albuminuria, although his eGFR remained normal. Ocular and audiological assessments at that time were normal.

In addition, the proband’s grandaunt (I-1), aged 80, reportedly had hematuria and/or proteinuria, but further clinical details were unavailable.

### Family B

**Figure 2:**
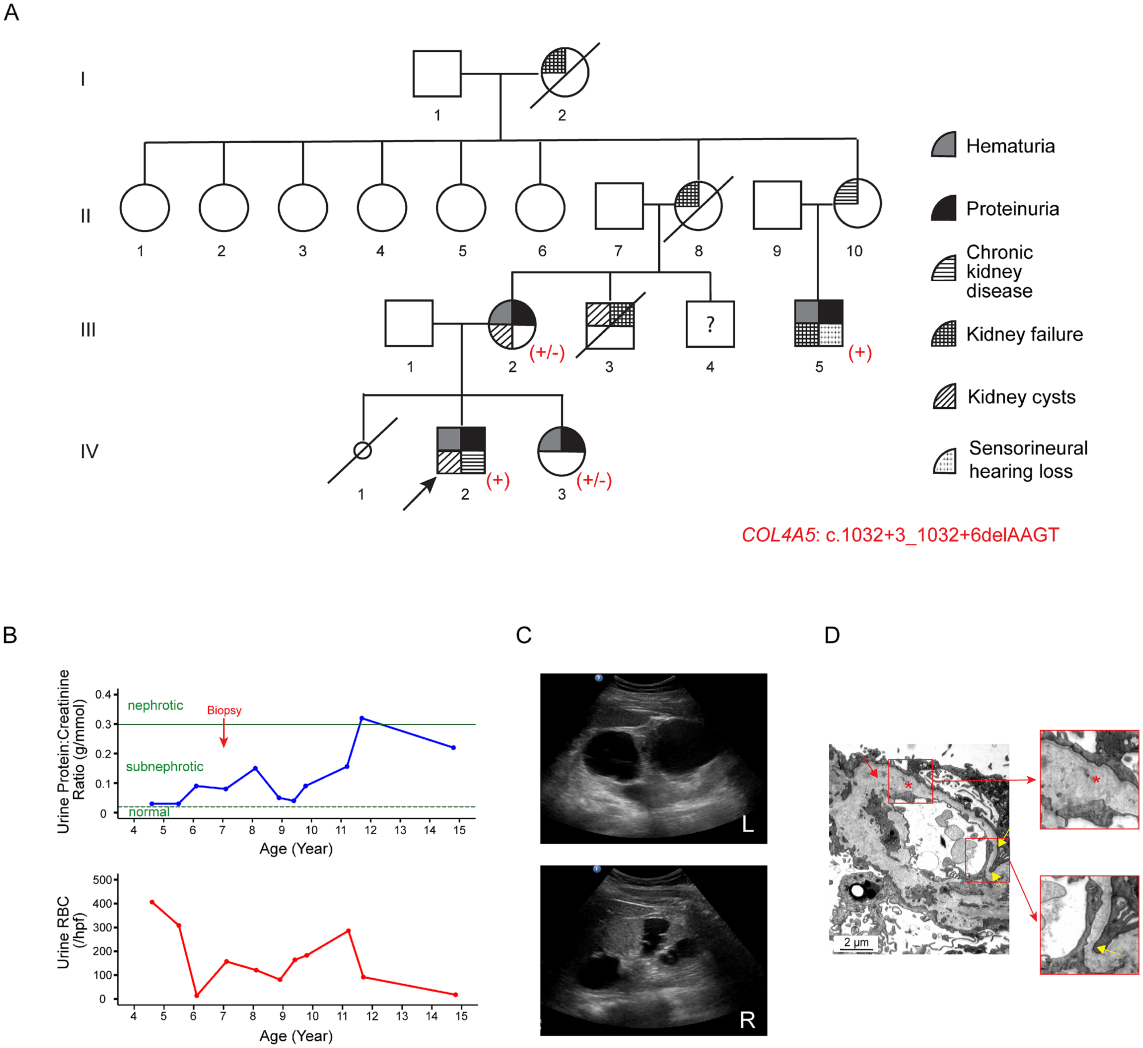
Clinical features of Family B associated with the variant c.1032 + 3_1032 + 6delAAGT in *COL4A5*. **(A)** Pedigree of the affected family. Family members are denoted by generations I-IV. The arrow marks the proband; a slash strikethrough indicates deceased individuals; the smaller circle denotes a stillborn female baby. Genotypes are shown as +/− for heterozygous female individuals and + for hemizygous male individuals carrying the variant. **(B)** Disease progression in the proband (IV-2). The urine protein-to-creatinine ratio (g/mmol) and urinary red blood cell count (RBCs/hpf) are displayed against age in years. The proband first presented at the age of three with recurrent synpharyngitic gross hematuria and was subsequently found to have persistent microscopic hematuria. Subnephrotic proteinuria was also noted. The green horizontal dotted and solid lines represent the cutoff values for sub-nephrotic (urine protein-to-creatinine ratio: 0.02 g/mmol) and nephrotic (urine protein-to-creatinine ratio: 0.3 g/mmol) proteinuria, respectively. **(C)** Kidney ultrasound of the proband at the age of 15. Multiple large bilateral kidney cysts were observed, with the largest cyst in the right kidney measuring 2.7 cm × 3.4 cm and the largest in the left kidney measuring 5.0 cm × 5.3 cm. Upper panel: left kidney; lower panel: right kidney. **(D)** Representative electron microscopy images from the kidney biopsy of the proband’s maternal uncle (III-5). The GBM featured alternating thickened segments (~769 nm to 1,244 nm) and thinning regions (~ 153 nm; yellow arrows and lower insert). The normal GBM thickness for a 15-year-old male individual ranges from 230 to 430 nm (15). The lamina densa displayed textural irregularities with some degree of lamellation and splitting (red asterisk and upper insert), suggestive of Alport syndrome.

The proband (IV-2; Figure 2A) first presented at the age of three with recurrent synpharyngitic gross hematuria and was subsequently found to have persistent microscopic hematuria (70 RBCs/hpf with 75% being dysmorphic). Subnephrotic proteinuria was noted, with a urine protein-to-creatinine ratio of 0.05 g/mmol. His eGFR was normal, and his autoimmune screen was negative. The patient was initially treated with cyclosporine A; however, this was discontinued due to the progressive decline in kidney function. Due to this, ACE-i therapy was commenced (Figure 2B). Notably, a kidney ultrasound performed at age 15 revealed bilateral echogenic kidneys, with the right and left kidneys measuring 10.7 cm and 13.8 cm in length, respectively. Multiple large bilateral kidney cysts were observed, with the largest cyst in the right kidney measuring 2.7 cm x 3.4 cm and the largest cyst in the left kidney measuring 5.0 cm x 5.3 cm (Figure 2C). Audiological assessments performed at the same age were normal. At present, aged 16, the proband’s kidney function has progressively deteriorated to KDIGO stage G4A3.

The proband’s mother (III-2) first presented at age 24 with persistent microscopic hematuria and proteinuria (Figure 2A). At age 26, she was found to have bilateral kidney cysts with septations. Kidney biopsy revealed focal segmental glomerulosclerosis (FSGS). During pregnancy, she developed pre-eclampsia, and an audiological evaluation at that time detected hearing loss. At the current age of 44, her eGFR remains within normal limits.

The proband’s sister (IV-3) was found to have persistent hematuria (up to 60 RBCs/hpf) and subnephrotic proteinuria (0.5 g/day/1.73m^2^) at the age of seven. She was initiated on ACE-i therapy, and her eGFR has remained within normal limits.

The proband’s distant maternal uncle (III-5), currently 28 years old, presented with persistent nephrotic-range proteinuria at age 15. At the initial presentation, his eGFR was 71 mL/min/1.73m^2^. Kidney biopsy was suggestive of Alport syndrome (see Histological examination below). He subsequently progressed to kidney failure in his third decade of life and underwent kidney transplantation. In addition, he had bilateral sensorineural hearing loss.

Other maternal family members, including the proband’s maternal uncle (III-3), grandmother (II-8), grandaunt (II-10), and maternal great-grandmother (I-2), had a history of chronic kidney disease and/or kidney failure requiring kidney replacement therapy.

## Light and electron microscopy examinations of the kidney samples

### Family A

A kidney biopsy was performed on the proband (III-3) at age 21 due to the presence of A3 albuminuria. A total of 30 glomeruli were examined. Light microscopy revealed a mild increase in the mesangial matrix without mesangial hypercellularity. Electron microscopy showed effacement of podocyte foot processes. The GBM exhibited areas of variable thinning and thickening, with occasional segments displaying lamination. Irregular lucencies were observed within the lamina rara interna, accompanied by scattered microparticles along the GBM.

The proband’s son (IV-2) underwent a kidney biopsy at age six following the development of nephrotic-range proteinuria. A total of 20 glomeruli were examined. Light microscopy showed generally normocellular glomeruli with patent capillary lumina and predominantly single-contoured capillary walls, although there were segments of ischemic wrinkling. Two glomeruli displayed small segments of accentuated wrinkling, albeit without established sclerosis. Electron microscopy revealed significant variability in GBM thickness, with alternating regions of thinning (~150 nm) and thickening (~457 nm), along with the characteristic “basket-weaving” appearance in the thickened region (Figure 1D) (Normal GBM thickness for a 6-year-old male individual ranges from 198 to 371 nm) (15). This lamellated and irregular morphology of the GBM is highly indicative of Alport syndrome.

### Family B

A kidney biopsy was performed on the proband (IV-2) at age seven when he had subnephrotic proteinuria with a normal eGFR. A total of 52 glomeruli were obtained. Light microscopic examination revealed generally normocellular glomeruli, with three glomeruli showing segmental mesangial hypercellularity. The capillary loops were patent, without the evidence of double-contoured capillary walls. There was focal mild tubular atrophy. Ultrastructural evaluation by electron microscopy showed diffuse and global thinning of the GBM, with basement membrane thickness across various capillary loops ranging from 122 nm to 201 nm (normal GBM thickness for 7-year-old male individuals ranges from 209 to 391 nm) (15). Podocyte foot process effacement was present, accompanied by microvillous transformation of the podocyte cytoplasm. These findings were consistent with thin GBM nephropathy.

The proband’s maternal uncle (III-5) underwent a kidney biopsy at age 15 when he had KDIGO stage G2A3. A total of 16 glomeruli were sampled. Light microscopy showed that most glomeruli displayed wrinkling of capillary loops and basement membranes with textural irregularities, double-contoured segments, and occasional vacuolations. Mild mesangial expansion with increased cellularity was also noted. Electron microscopy showed the GBM with alternating thickened segments (~769 nm to 1,244 nm) and thinning portions (~153 nm to 268 nm) (normal GBM thickness for a 15-year-old male individual ranges from 230 to 430 nm) (15). In addition, the lamina densa exhibited textural irregularities with areas of lamellation and splitting (Figure 2D).

## Exome sequencing of the probands

Genomic DNA was extracted from leukocytes, and exome sequencing was performed. The results revealed a heterozygous variant in *COL4A5* [NM_000495.5]: c.1032 + 4A > G in the proband of Family A (III-3) and a hemizygous four-nucleotide deletion in *COL4A5* [NM_000495.5]: c.1032 + 3_1032 + 6delAAGT in the proband of Family B (IV-2). The variants are located within intron 18 of the *COL4A5* gene, proximal to the boundary between exon 18 and intron 18. Both variants are absent in population databases (Genome Aggregation Database; gnomAD v4.1.0). As shown in Supplementary Table S1, the variant *COL4A5*: c.1032 + 3_1032 + 6delAAGT is predicted to strongly disrupt the donor site, with scores of 1.00 and 0.86 and a shift from 8 to −19.72 by SpliceAI (16), Pangolin (17), and MaxEnt (18, 19), respectively. In contrast, the variant *COL4A5*: c.1032 + 4A > G is predicted to moderately affect splicing, with scores of 0.46 and a shift from 8 to 2.94 by Pangolin and MaxEnt, respectively. This evidence suggests that these variants, located at non-canonical splice sites, are predicted to disrupt splicing by breaking the natural splice donor motif.

## Familial segregation of the genetic variants

To further evaluate the pathogenicity of the variants, a detailed pedigree tree was drawn and the phenotypes for the family members were obtained where possible. Sanger sequencing for the variant *COL4A5*: c.1032 + 4A > G was performed in Family A for individuals III-1 (sister) and IV-2 (son). The variant was detected in IV-2, who had progressed to KDIGO stage G2A2 by the age of 9 years, but it was absent in III-1, who exhibited no clinical evidence of kidney disease (Figure 1A). Similarly, Sanger sequencing for the variant *COL4A5*: c.1032 + 3_1032 + 6delAAGT was performed in Family B for members III-2 (mother), III-5 (maternal uncle), and IV-3 (sister). This variant was identified in all three family members, each displaying varying degrees of kidney disease severity (Figure 2A).

## Immunofluorescence analysis of the kidney samples

**Figure 3:**
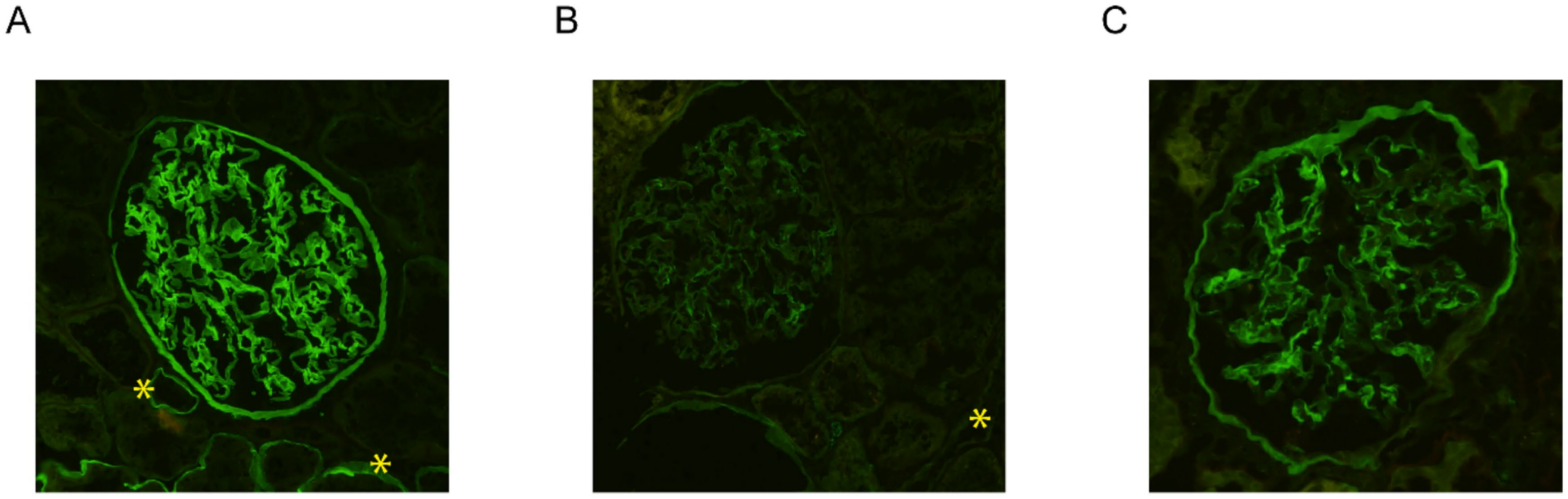
Immunofluorescence staining for collagen IV α5 in the kidney biopsy samples. While the control kidney tissue exhibited uniform, continuous staining of collagen IV α5 across the glomerular basement membrane (GBM), distal tubular basement membrane (TBM), and Bowman’s capsule **(A)**, immunofluorescence staining of the kidney tissue from the proband’s son in family A (IV-2) demonstrated an interrupted, mosaic staining pattern along the GBM and TBM, with significantly diminished or absent staining in Bowman’s capsule **(B)**. Immunostaining analysis of the kidney tissue from the proband in family B (IV-2) exhibited an abnormal mosaic and mildly diminished pattern along the GBM and Bowman’s capsule, with a complete absence of staining along the distal TBM **(C)**. Distal TBMs are marked with yellow asterisks. All three images were taken at 200x magnification.

In view of the electron microscopy changes seen in the kidney sample of the proband’s son (IV-2) in Family A, immunofluorescence staining for collagen IV α5 was performed. This demonstrated interrupted, mosaic staining patterns along the GBM and distal tubular basement membrane (TBM), with significantly diminished or absent patterns in Bowman’s capsule (Figure 3B). In contrast, control kidney tissue exhibited uniform, continuous staining of collagen IV α5 across the GBM, TBM, and Bowman’s capsule (Figure 3A).

Similarly, immunofluorescence staining for collagen IV α5 was performed on the kidney tissue of the proband (IV-2) in Family B. This exhibited an abnormal mosaic and mildly diminished pattern along the GBM and Bowman’s capsule, with a complete absence of staining along the distal TBM (Figure 3C). Immunofluorescence for collagen IV α5 in the proband’s maternal uncle (III-5) from Family B also showed absence of staining in the GBM, Bowman’s capsule, and distal TBM.

## Minigene splicing assay

**Figure 4:**
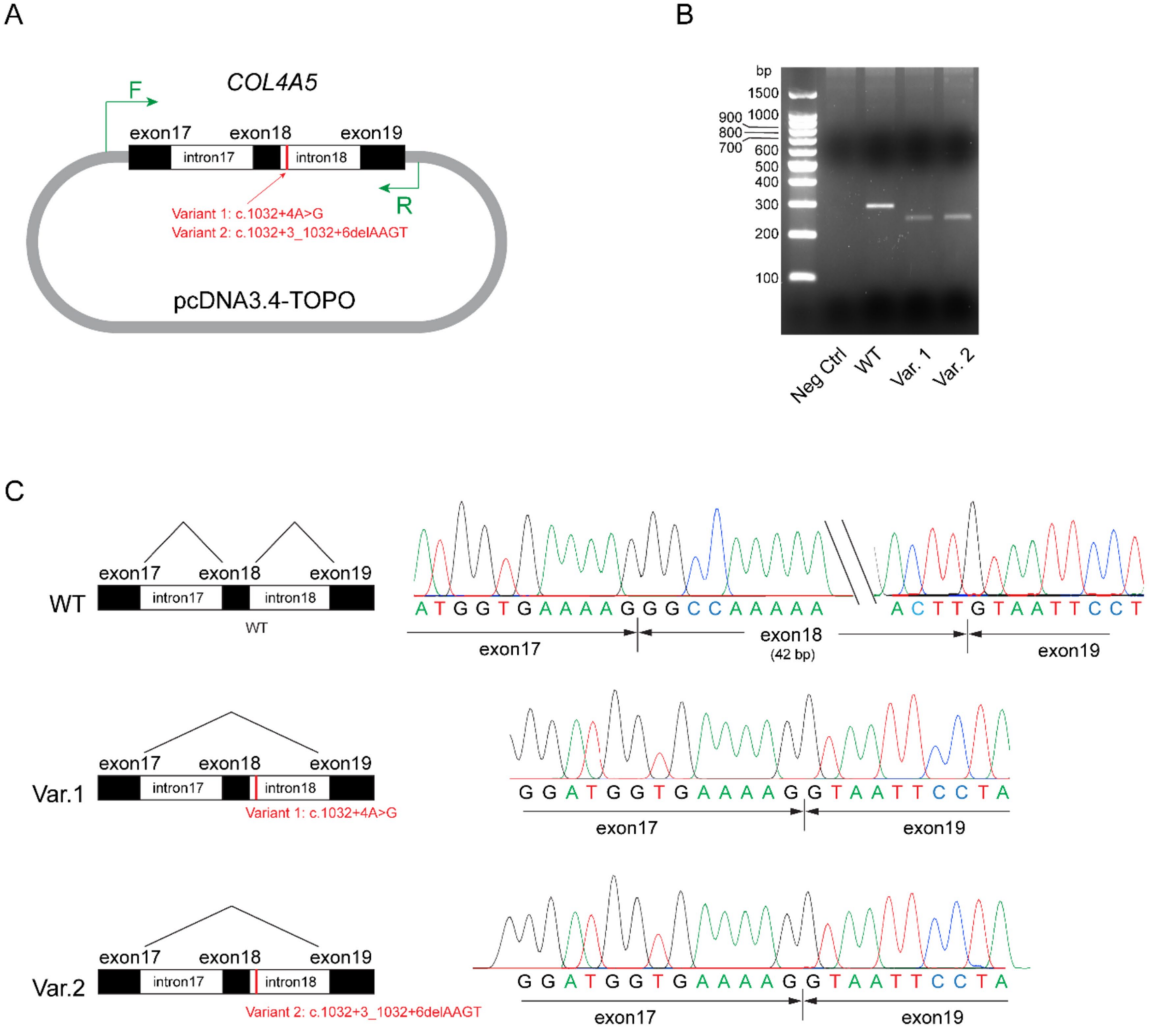
Minigene assay for variants *COL4A5*: c.1032 + 4A > G and *COL4A5*: c.1032 + 3_1032 + 6delAAGT. **(A)** Schematic of the plasmid structures. Exons 17 to 19, together with introns 17 and 18 of the wild-type *COL4A5* gene (hg38: chrX-108,582,886-108,586,715), were amplified and ligated into the pcDNA3.4-TOPO vector. The mutant variants (Variant 1: c.1032 + 4A > G and Variant 2: c.1032 + 3_1032 + 6delAAGT) were separately introduced into the control plasmids through site-directed mutagenesis. The short vertical red line marks the variant loci, while the green arrows indicate primer pairs targeting vector-specific regions for polymerase chain reaction (PCR) amplification. **(B)** Agarose gel analysis of transgene transcripts. Total RNA was extracted from transfected HEK293 cells and reverse transcribed into cDNA. Transgene transcripts were amplified using primers targeting vector-specific regions, with cDNA as the template. While the negative control (pcDNA3.4-TOPO vector) did not yield any observable bands on the agarose gel (lane 2), the plasmid containing the wild-type (WT) form of exons 17 to 19 of *COL4A5* produced an intense band approximately 300 bp in size (lane 3). Plasmids harboring Variants (Var) 1 and 2 generated truncated transcripts, as evidenced by faint bands ranging from 200 bp to 300 bp (lanes 4 and 5). **(C)** Sanger sequencing results of the PCR products. Transcripts from the plasmid carrying the wild-type (WT) sequence included exons 17, 18, and 19 (top panel), whereas transcripts from plasmids carrying Variants 1 and 2 exhibited the skipping of exon 18 (middle and bottom panels, respectively).

A minigene splicing assay was conducted to evaluate the impact of these non-canonical splice site variants on pre-messenger RNA (mRNA) splicing and transcriptional outcomes. Briefly, the wild-type (WT) genomic sequence of *COL4A5* (hg38: chrX-108,582,886-108,586,715), encompassing exons 17 to 19 and flanking introns 17 and 18, was amplified from the peripheral blood leukocytes of a healthy individual and subsequently cloned into the pcDNA3.4 expression vector to generate a wild-type control plasmid. The two mutant constructs were created by introducing the respective variants into the wild-type plasmid via site-directed mutagenesis (Figure 4A). The constructed plasmids were transfected into HEK293 cells, and total RNA was extracted 4 h post-transfection. Reverse-transcribed complementary DNA (cDNA) was used as a template for the amplification of transgene-derived transcripts with vector-specific primers. The wild-type construct yielded an intense band approximately 300 bp in size (Figure 4B). Conversely, the mutant plasmids harboring either variant generated a truncated transcript. Sanger sequencing of the purified polymerase chain reaction (PCR) products confirmed that the transcripts derived from the mutant plasmids exhibited exon 18 skipping (Figure 4C). Collectively, these findings demonstrate that both splice site variants lead to aberrant splicing, thereby confirming their functional impact.

## Classification of the variants

Variant interpretation and classification were conducted in accordance with the guidelines of the ACMG/AMP (14, 20). These criteria were subsequently converted into quantitative point values (21) and integrated into a Bayesian framework for variant interpretation (22). The minigene splicing assay demonstrated that both variants caused aberrant splicing, leading to the skipping of exon 18, thereby fulfilling the criterion for PVS1_strong (23). When considered alongside additional lines of evidence, including minor allele frequencies from population databases, specific phenotype features such as pathological findings, and co-segregation among the family members, both variants met the threshold for reclassification as “Pathogenic” under the ACMG/AMP guidelines (see Supplementary Results and Supplementary Table S2).

## Discussion

Interpretation of VUSs is a challenge in clinical genomic medicine, and this problem is exacerbated in the Asian population, which is underrepresented in genomic population-based databases (9). In this study, we demonstrated the use of the minigene assay, in conjunction with other lines of evidence such as immunofluorescence analysis of kidney biopsies and familial segregation, to upgrade two non-canonical splice VUSs in the *COL4A5* gene to ‘Pathogenic’ based on the ACMG / AMP criteria. Functionally, we demonstrated that both VUSs within intron 18 of the *COL4A5* gene, c.1032 + 4 A > G and c.1032 + 3_1032 + 6delAAGT, can result in the skipping of exon 18 and consequently create a transcript with a 42-bp deletion.

Splicing is the process of removing RNA sequences transcribed from intronic DNA regions and joining the remaining exonic regions to form mRNA, which is required for protein synthesis. This process requires proper intron recognition and spliceosome assembly and is facilitated by conserved regulatory sequences located at the intron/exon boundaries. The majority of introns are flanked by a GT dinucleotide at the 5′ end and an AG dinucleotide at the 3′ end (GT-AG). These are known as canonical splice sites at the intron/exon boundaries.

Disruptions in pre-mRNA splicing are an important pathomechanism in genetic disease—it is estimated that one-third of pathogenic variants cause aberrant splicing (24). Canonical splice site variants have been categorized as “very strong” diagnostic candidates in disorders where loss of function is a known disease mechanism (14). The role of variants in non-canonical splice sites, such as deep intronic variants or substitutions in exons, is also increasingly recognized. Exonic variants can interfere with splicing regulatory elements, resulting in exon skipping, while intronic variants can result in cryptic splice sites or cause retention of intronic fragments (25, 26). In exome sequencing data, 27% of pathogenic splicing variants are non-canonical. In XLAS, up to 18% of pathogenic *COL4A5* variants are splicing variants, and non-canonical splicing variants have also been reported (26).

In addition to the non-detection of deep intronic splice site variants by conventional sequencing techniques, clinical interpretation of non-canonical splice site variants is also challenging because there is often the need to demonstrate aberrant splicing as an additional piece of evidence to prove pathogenicity. Hence, transcriptional analysis is often required to facilitate re-classification of these variants.

Variant interpretation is highly variable, despite the availability of standard ACMG/AMP guidelines. For example, the variant *COL4A5*: c.1032 + 4A > C described in Family B has been previously reported in a 36-year-old male patient (27). This was classified as “pathogenic or likely pathogenic” based on the criteria of PP3_Very Strong and PM2_Supporting, although these two pieces of evidence may not be enough for one to designate a “Likely Pathogenic” criterion based on the ACMG/AMP guidelines and recent Bayesian framework (14, 20, 22). In ClinVar, this same variant (Variation ID: 2616665) was classified as a VUS (single submission). The variant *COL4A5*: c.1032 + 3_1032 + 6delAAGT found in Family A has been reported in a 10-year-old male patient with chronic kidney disease stage 2 (28), a patient with kidney failure at 26 years old (29), and a 12-year-old male patient with no further phenotype details (30). Consistent with our results, the authors of the latter study showed that this variant causes exon 18 skipping. The vector expression system in the minigene assay of our study is different from the one previously reported. Since the variant (c.1032 + 4A > G) has never been studied before, both variants were included in our minigene assay. As both variants are located within the same splicing motif, incorporating the variant c.1032 + 3_1032 + 6delAAGT as “positive control” served to provide an internal benchmark.

The ideal way to functionally characterize a VUS predicted to involve splice sites is to conduct *in vivo* transcriptional analysis using the patients’ affected tissues to examine tissue-specific gene expression (13). In the case of Alport syndrome, kidney biopsy samples are often not readily available for such studies. Alternative options include *in silico* splice prediction, but the sensitivity of this method is low (31). Transcription assays, such as the minigene assay, are useful for investigating aberrant splicing because they do not require tissue-specific biological samples and their results can be consistent with *in vivo* studies (11, 13, 30). Nevertheless, routine clinical use of transcription assays may be limited due to a lack of widespread expertise and the labor-intensive nature of these procedures.

Moreover, kidney cysts are increasingly reported in individuals with pathogenic variants in *COL4A3-COL4A5* and a diagnosis of Alport syndrome (3). These cysts, often appearing before age 50, are typically confined to the kidneys and are more commonly found in individuals with proteinuria, suggesting a link to more severe disease (4). While the kidney cysts are not large enough to cause direct renal decline, their presence may indicate disease progression. Recent studies have reported cysts in approximately 38% of genetically confirmed patients with Alport syndrome, with some showing increased kidney volume that can mimic autosomal dominant polycystic kidney disease (ADPKD) (5). As highlighted in the latest ERKNet guidelines (32), genetic testing for cystic kidney disease should include *COL4A3-COL4A5* to ensure accurate diagnosis and avoid misclassification.

We also described the use of immunofluorescence analysis for collagen IV α5 in kidney tissues to substantiate the interpretation of VUSs. While classical features of electron microscopy of Alport syndrome can be adequate for a clinical diagnosis of Alport syndrome, such findings are often not present in the earlier stages of the disease (31, 33, 34). On the other hand, approximately 70–80% of male individuals with X-linked Alport syndrome exhibit absent staining for collagen IV α5 chains (35). Consequently, immunofluorescence staining for type IV collagen can be invaluable for diagnostic purposes. The abnormal immunostaining of collagen IV α5 may further support the evidence that these families carry phenotypes specific to this disease.

In conclusion, we demonstrate a structured evaluation approach for the reclassification of two previously reported VUSs by applying multiple ACMG /AMP guidelines, in accordance with the Bayesian framework. By implementing this VUS resolution program, we can systematically resolve variants with uncertain significance and increase diagnostic yield in genomic medicine within nephrology (36).

## Data Availability

The original contributions presented in the study are included in the article/Supplementary material; further inquiries can be directed to the corresponding authors.
