# Supplementary material for: Case Report: Evaluation of COL4A5 non-canonical splicing variants in two families
Source: Front Med (Lausanne). 2025 Sep 29;12:1611334. doi: 10.3389/fmed.2025.1611334 (PMC12515852; doi:10.3389/fmed.2025.1611334)
Supplement: Supplementary file 1 [file Data_Sheet_1.pdf]

Supplementary Materials

Evaluation of *COL4A5* Non-canonical Splicing Variants in Two Families

Contents

Supplementary Methods 2
Electron microscopy 2
Immunofluorescence staining 2
Minigene Assay 2
Supplementary Results 5
Variant Classification 5
Tables 7
References 9

Supplementary Materials

Supplementary Methods

Electron microscopy

Renal biopsies were fixed in glutaraldehyde overnight and subsequently fixed with 1% osmium tetroxide for 1 hour. The specimens were then dehydrated in an ascending acetone series, embedded in epoxy resin and polymerized for 12 hours at 70°C. Ultrathin sections were prepared and contrasted with uranyl acetate and lead citrate and subsequently examined using a transmission electron microscope JEM-2100 (JEOL, Japan). Images were taken with Gatan Orius SC200 camera (Gatan Inc, USA).

Immunofluorescence staining

Frozen sections were obtained from fresh renal biopsy and stained with FITC- Anti Collagen IV alpha 5 antibody (Cosmo Bio Co. Ltd, Japan). Images were captured using DP74 digital camera (Olympus, Japan).

Minigene Assay

<u>Plasmid construction</u>

Polymerase chain reaction (PCR) was performed to generate a 3.8 kb fragment encompassing exons 17 to 19 and introns 17 and 18 of the *COL4A5* gene from the genomic DNA of healthy control. The primers used for this amplification were TTT GCC TGG TGA TCC TGG TTA C (forward) and ATT CCA GGA AAT CCT CGC TCT (reverse). The composition of each PCR reaction mixture included: 5 μL of 10x high fidelity buffer, 2 μL of 50 mM $MgSO_4$, 1 μL of 10 mM dNTPs, 1 μL of 10 μM primers, 1 μL of genomic DNA, and 0.2 μL of Platinum Taq DNA Polymerase (Invitrogen, Thermo Fisher Scientific), totalling a 50 μL reaction volume. The PCR cycling protocol was structured in three phases: (1) an initial denaturation at 94°C for 1 minute, (2) 26 cycles of 94°C for 15 seconds, 58°C for 30 seconds, and 68°C for 30 seconds, and (3) a final elongation at 68°C for 2 minutes. The integrity of the PCR product was verified by electrophoresis on a 1% agarose gel to ensure the presence of a single PCR product.

Supplementary Materials

PCR products were purified using the Monarch PCR and DNA Cleanup Kit (New England Biolabs) and subsequently ligated into the pcDNA3.4-TOPO-TA vector employing the TOPO TA Cloning Kit (Invitrogen, Thermo Fisher Scientific), following the manufacturer's guidelines. Plasmids were produced using One Shot TOP10 Chemically Competent *E*. coli (Invitrogen, Thermo Fisher Scientific) and extracted with the Monarch Midi Prep Kit (New England Biolabs), adhering to the provided protocols. The variants *COL4A5*: c.1032+4A>G and c.1032+3_1032+6delAAGT were introduced into the plasmid through direct nucleotide synthesis conducted by Azenta US Inc.

Plasmid sequences were verified through nanopore plasmid sequencing to ensure accurate insertion and mutation of the variant sequences.

Cell culture and transfection

HEK293 cells were maintained at 37°C in an atmosphere of 5% CO_2, in Dulbecco's Modified Eagle Medium (DMEM) supplemented with 10% fetal bovine serum and 1% Penicillin-Streptomycin. One day prior to transfection, cells were seeded into 3.5 cm Petri dishes. Upon reaching approximately 70% confluency, 2 μg of plasmid DNA were transfected into each dish using 4 μL of jetPRIME transfection reagent (Polyplus) following the manufacturer's protocol. Cells were harvested four hours post-transfection for RNA extraction and reverse-transcription.

Reverse-transcript PCR

Total RNA was extracted from transfected cells utilizing the Monarch Total RNA Miniprep kit (New England Biolabs) and quantified via a Nanodrop spectrophotometer. Reverse transcription was conducted using 2 μg of each RNA sample with the Maxima First Strand cDNA Synthesis system (Thermo Fisher Scientific) in a 20 μL reaction volume, adhering to the manufacturer's protocol. PCR amplification was subsequently performed using vector-specific primers: GTT TTG ACC TCC ATA GAA GAC A (forward) and CAT TAC TAA CCG GTA GGG ATC (reverse). The 50 μL reaction mixture comprised 2.5 μL of cDNA, 5 μL of 10X High Fidelity buffer, 1 μL of 10 μM of each primer, 1 μL of 10 mM dNTPs, and 0.25 μL of Platinum High Fidelity Taq DNA polymerase. PCR cycling conditions were as follows: an initial denaturation at 94°C for 1 minute, followed by 26 cycles of 94°C for 15 seconds, 55°C for 30 seconds, and 68°C for 30 seconds, with a final elongation step at 68°C for 2 minutes. PCR products were resolved by gel electrophoresis

on a 2% agarose gel stained with RedSafe nucleic acid staining solution, alongside a 100 bp DNA ladder.

Sanger Sequencing

PCR products were purified using the Monarch PCR and DNA Cleanup Kit (New England Biolabs) and Sanger sequences were performed by Axil Scientific Pte. Ltd using primers GTT TTG ACC TCC ATA GAA GAC A (forward) and CAT TAC TAA CCG GTA GGG ATC (reverse).

Supplementary Materials

Supplementary Results

Variant Classification

This is based on ACMG/AMP guidelines [12, 17], and the Bayesian framework [19].

<u>*COL4A5*: c.1032+4A>G</u>

PVS1_strong (4 *pt*): The variant induces an alternative splicing product characterized by the skipping of exon 18. According to the PVS1 decision tree, the variant is classified as follows: “Deletion (single exon to full gene)”→ “Single to multi exon deletion – Preserves reading frame”→ “Truncated/altered regions is critical to protein function”→ “PVS1_strong” (1).

PM2_supporting (1 *pt*): The variant was absent in population databases.

PP4_supporting (2 *pt*): The patient demonstrated characteristic phenotypes of Alport syndrome, including immunofluorescence staining and electron microscopy analysis of kidney biopsy samples. Given that pathogenic variants in the *COL4A5* gene are implicated in approximately one-third of Alport syndrome cases, 2 points were assigned to PP4 (2).

PP1_moderate (2 *pt*): The variant exhibited co-segregation with the disease across multiple affected family members. Specifically, the proband III-3 had one affected relative IV-2 and one unaffected relative III-1. Consequently, 2 points were assigned.

A total of 4 points were assigned to PP1 + PP4 (2 *pt* + 2 *pt* = 4 *pt*) (2).

PS1_moderate (2 *pt*): Our minigene has proven this variant results in exon 18 skipping. This exon 18 skipping has resulted in the same transcript that is also shown in the variant COL4A5: c.1032+3_1032+6delAAGT, which has been classified as "Pathogenic" by Japanese group. Therefore, PS1_moderate (2 pt) was assigned (3).

In total: 4 *pt* + 1 *pt* + 4 *pt* +2 *pt* = 11 *pt* → **Pathogenic**

Supplementary Materials

COL4A5: c.1032+3_1032+6delAAGT

PVS1_strong (4 *pt*): The variant induces an alternative splicing product characterized by the skipping of exon 18. According to the PVS1 decision tree, the variant is classified as follows: "Deletion (single exon to full gene)"→ "Single to multi exon deletion – Preserves reading frame"→ "Truncated/altered regions is critical to protein function"→ "PVS1_strong" (1).

PM2_supporting (1 *pt*): The variant was absent in population databases.

PP4_supporting (2 *pt*): The patient exhibited characteristic Alport syndrome phenotypes, including mosaic immunohistochemistry staining for collagen IV α5 in kidney biopsies, and variable alterations in glomerular basement membrane (GBM) thickness, with regions of significant thinning (approximately 150 nm) and thickening (approximately 460 nm). Thickened segments of the GBM exhibited a disorganized, mottled appearance, often described as basket-weaving. Given that pathogenic variants in the *COL4A5* gene are implicated in approximately one-third of Alport syndrome cases, 2 points were assigned to PP4 (2).

PP1_strong (5 *pt*): The variant exhibited co-segregation with the disease across multiple affected family members. Specifically, the proband IV-2 had five affected relatives who also carried the variant: II-8 (obligate carrier), II-10 (obligate carrier), III-2, III-5, and IV-3. Consequently, 5 points were assigned.

A total of 5 points were assigned to PP1 + PP4 (5 *pt* + 3 *pt* = 8 *pt*; capped at 5 *pt*) (2).

In total: 4 *pt* + 1 *pt* + 5 *pt* = 10 *pt* → **Pathogenic**

Supplementary Materials

Tables

Table S1. Splicing effects of the two variants predicted by multiple computational tools.

	NM_000495. 5(*COL4A5*): c.1032+4A>G		NM_000495. 5(*COL4A5*): c.1032+3_1032+6delAAGT	
	**Type**	**Score**	**Type**	**Score**
**SpliceAI**	Acceptor Loss	0.20	Acceptor Loss	0.97
	Donor Loss	0.00	Donor Loss	1.00
**Pangolin**	Splice Loss	0.46	Splice Loss	0.86
	Splice Gain	0.04	Splice Gain	0.01
**HSF**	Broken Donor Sites	83.44 to 73.43	Broken Donor Sites	-
**MaxEnt**	Broken Donor Sites	8 to 2.94	Broken Donor Sites	8 to -19.72

Supplementary Materials

Table S2. Comparison of variant interpretation and classification before and after to this study with or without minigene application.

Variants	Variant interpretation and classification prior to this study	Variant interpretation and classification after this study[b] before minigene application	Variant interpretation and classification after this study[b] after minigene application
COL4A5 (NM_000495.5): c.1032+4A>G	VUS – PM2_supporting (PMID 37915894) PP4_supporting (PP3 not applied)[a]	VUS – PM2_supporting PP4_supporting **PP1_moderate**	Pathogenic – PM2_supporting PP4_supporting **PP1_moderate** **PVS1_strong** **PS1_moderate**
COL4A5 (NM_000495.5): c.1032+3_1032+6delAAGT	Likely pathogenic – PVS1_strong (PMID 29959198) PM2_supporting (Varsome, PMID 39625990) PP4_supporting	Pathogenic – PVS1_strong PM2_supporting PP4_supporting **PP1_strong**	Pathogenic – PVS1_strong PM2_supporting PP4_supporting

[a] PP3_Very Strong was not assigned because variants located at potential splice sites should first be determined using in silico tools (e.g. SpliceAI) if they can affect splicing, and this prediction result should be used for PVS1 decision tree. In silico prediction tools for protein functional defects (e.g. REVEL, CADD scores) are for PP3 criteria and should be considered only if the splicing may result in functional protein defects.

[b] For justification of the ACMG criteria, please refer to the section above "Variant Classification".

Codes in bold are added as the result of this study.

Supplementary Materials

References

1. Abou Tayoun AN, Pesaran T, DiStefano MT, Oza A, Rehm HL, Biesecker LG, et al. Recommendations for Interpreting the Loss of Function Pvs1 Acmg/Amp Variant Criterion. *Hum Mutat* (2018) 39(11):1517-24. Epub 20180907. doi: 10.1002/humu.23626.
2. Biesecker LG, Byrne AB, Harrison SM, Pesaran T, Schaffer AA, Shirts BH, et al. Clingen Guidance for Use of the Pp1/Bs4 Co-Segregation and Pp4 Phenotype Specificity Criteria for Sequence Variant Pathogenicity Classification. *Am J Hum Genet* (2024) 111(1):24-38. Epub 20231215. doi: 10.1016/j.ajhg.2023.11.009.
3. Walker LC, Hoya M, Wiggins GAR, Lindy A, Vincent LM, Parsons MT, et al. Using the Acmg/Amp Framework to Capture Evidence Related to Predicted and Observed Impact on Splicing: Recommendations from the Clingen Svi Splicing Subgroup. *Am J Hum Genet* (2023) 110(7):1046-67. Epub 20230622. doi: 10.1016/j.ajhg.2023.06.002.
